# The Importance of Therapeutically Targeting the Binary Toxin from *Clostridioides difficile*

**DOI:** 10.3390/ijms22062926

**Published:** 2021-03-13

**Authors:** Dinendra L. Abeyawardhane, Raquel Godoy-Ruiz, Kaylin A. Adipietro, Kristen M. Varney, Richard R. Rustandi, Edwin Pozharski, David J. Weber

**Affiliations:** 1 Department of Biochemistry and Molecular Biology, University of Maryland School of Medicine, Baltimore, MD 21201, USA; dabeyawardhane@som.umaryland.edu (D.L.A.); rruiz@som.umaryland.edu (R.G.-R.); kadipietro@som.umaryland.edu (K.A.A.); kvarney@som.umaryland.edu (K.M.V.); epozharskiy@som.umaryland.edu (E.P.); 2 Baltimore—Institute for Bioscience and Biotechnology Research, University of Maryland-Institute for Bioscience and Biotechnology Research, Rockville, MD 20850, USA; 3 The Center for Biomolecular Therapeutics, University of Maryland School of Medicine, Baltimore, MD 21201, USA; 4 Merck & Co., Inc., Kenilworth, NJ 07033, USA; richard_rustandi@merck.com

**Keywords:** *Clostridioides difficile*, binary toxin, infectious disease, protein structural biology, CDT, CDTa, CDTb

## Abstract

Novel therapeutics are needed to treat pathologies associated with the *Clostridioides difficile* binary toxin (CDT), particularly when *C. difficile* infection (CDI) occurs in the elderly or in hospitalized patients having illnesses, in addition to CDI, such as cancer. While therapies are available to block toxicities associated with the large clostridial toxins (TcdA and TcdB) in this nosocomial disease, nothing is available yet to treat toxicities arising from strains of CDI having the binary toxin. Like other binary toxins, the active CDTa catalytic subunit of CDT is delivered into host cells together with an oligomeric assembly of CDTb subunits via host cell receptor-mediated endocytosis. Once CDT arrives in the host cell’s cytoplasm, CDTa catalyzes the ADP-ribosylation of G-actin leading to degradation of the cytoskeleton and rapid cell death. Although a detailed molecular mechanism for CDT entry and host cell toxicity is not yet fully established, structural and functional resemblances to other binary toxins are described. Additionally, unique conformational assemblies of individual CDT components are highlighted herein to refine our mechanistic understanding of this deadly toxin as is needed to develop effective new therapeutic strategies for treating some of the most hypervirulent and lethal strains of CDT-containing strains of CDI.

## 1. Introduction

More than 2.8 million people were infected in 2019 by antibiotic-resistant bacteria in the United States alone, causing over 35,000 deaths [1]. The continued emergence of antibiotic-resistance demands that healthcare systems have both preventative and therapeutic measures available to counter this serious problem. Approximately twenty antibiotic-resistant bacteria were categorized by the Center for Disease Control (CDC) as urgent, serious, or concerning, based on the level of risk to human health with those most affected being the elderly, those with weakened immune systems, and/or those receiving prolonged antibiotic treatment(s) [2]. Considered here is one of the five most urgent infections recognized from the CDC from *Clostridioides difficile* (*C. difficile*), previously known as *Clostridium difficile* [1]. *C. difficile* is a gram-positive anaerobic pathogen responsible for antibiotic-associated diarrhea and pseudomembranous colitis caused by reduced levels of symbiotic gut microbiota [2,3]. The transmission of this disease occurs primarily in the form of highly stable spores, via the fecal-oral route, and is highly prevalent in hospital and nursing home settings [1,2].

**Figure 1:**
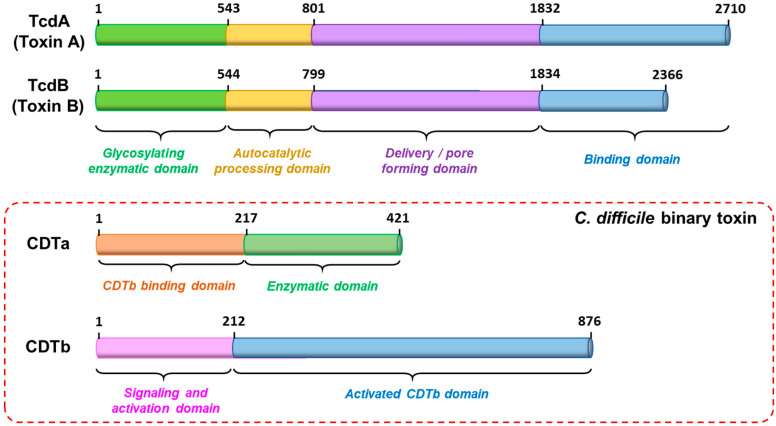
Schematic representation of the AB toxins causing *C. difficile* infection. The large enterotoxins (TcdA/Toxin A and TcdB/Toxin B) are composed of an N-terminal glycosylating enzymatic domain (green), an autocatalytic processing domain (yellow), a delivery and/or pore-forming domain for translocation (purple), and a binding domain with combined repetitive oligopeptides known as ‘CROPs’ (blue). The binary toxin, *C. difficile* transferase (CDT), consists of two independently produced components, CDTa and CDTb. The N-terminal domain of the enzymatic component (CDTa) binds to the binding component (CDTb) while the C-terminal domain of CDTa causes toxic ADP-ribosyltransferase activity within the host cell.

CDI is responsible for approximately 12,800 fatal deaths per year in the United States [1]. Severe CDI toxicity is associated with the “large” clostridial toxins, TcdA (toxin A) and TcdB (toxin B), and more recently from a potent binary toxin, CDT, identified for the first time early in the 21st century in hypervirulent strains [4]. TcdA and TcdB are classified together as AB toxins, consisting of an enzymatic subunit A and a delivery subunit B (Figure 1, top). The enzymatic subunit is an N-terminal glucosyltransferase domain responsible for disorganizing the intestinal epithelial cells by glycosylation of proteins from the Rho and Ras subfamilies [5]. In addition to the major toxins, 5–30% of clinical *C. difficile* isolates generate the binary toxin termed *C. difficile* transferase (CDT), which is associated with increased morbidity and mortality rates [6,7,8]. CDT was identified first in the *C. difficile* strain CD196 that was isolated from a patient with severe pseudomembranous colitis [7,8]. Unlike the contiguous polypeptide chain identified for the large clostridial toxins, the binary toxin is composed of two independently secreted A and B protein subunits (Figure 1, bottom). Therefore, in addition to targeting Tcda and Tcdb, the molecular mechanisms, giving rise to host cell toxicity from the binary toxin, CDT, require further study as needed to develop novel and effective therapies to prevent and/or provide treatment for this deadly bacterial infection [3].

## 2. CDT Epidemiology

Genotyping toxins of CDI is achieved using a PCR-restriction fragment length polymorphism (RFLP)-based method and is one method used to classify *C. difficile* strains into what is termed as toxinotypes [9]. In this regard, the differentiation of one *C. difficile* strain versus another is achieved by identifying changes in the pathogenicity locus (PaLoc), a 19 kb region coding for the toxin A (*tcdA*) and B (*tcdB*) gene transcripts. The PaLoc locus also encodes three accessory genes (*tcdR*, *tcdE*, and *tcdC*), which produces gene products that regulate toxins A and B expression. Over thirty toxinotype strains of CDI were identified in addition to a reference strain, VPI 10463, also termed toxinotype 0 [10]. Coincidently, the variant toxinotypes correlate well with PCR ribotypes, which are based on an rRNA phylogenetic typing, and represents an alternative method for classifying *C. difficile* strains.

*C. difficile* strains have been discovered with all combinations of toxins A, B, and CDT (A^+/−^, B^+/−^, and CDT^+/−^). However, the binary toxin is often not identified when testing using the toxinotype 0 reference method since CDT occurs most often in non-toxinotype 0 strains. Thus, detecting the *C. difficile* binary toxin is best achieved by testing directly for a 6.2 kb CdtLoc region that encodes the two CDT toxin genes (*cdtA* and *cdtB*) and its regulatory gene (*cdtR*) [10]. While the binary toxin-expressing strains were isolated first as a subset of *C. difficile* A^+^ and/or B^+^ strains, CDT expression (CDT^+^) can occur in A^−^/B^−^ strains of CDI (A^−^/B^−^/CDT^+^), and importantly, this strain displays CDI clinical phenotypes [11,12]. Although, the most well-studied binary toxin-containing strain is the *C. difficile* human epidemic strain, PCR ribotype 027 or toxinotype III (027/III), which expresses CDT and the A/B toxin (A^+^/B^+^/CDT^+^) [13]. Coincidently, the *C. difficile* strain CD196 also belongs to the PCR ribotype 027 and other epidemiological studies such as pulse-field gel electrophoresis (PFGE) and restriction endonuclease analysis (REA) recognize this *C. difficile* strain as type NAP1 and group BI, respectively. Therefore, the most often referenced CDT-containing strain of CDI is collectively referred to as the 027/B1/NAP1 strain. There are also several other prevalent toxinotypes isolated from humans from different continents. For instance, 027/III and 078/V are predominant in the United States and Europe, while 017/VIII and 244/IXb are variants most often identified in Asia and Australia, respectively [9]. For these reasons, understanding the toxinotypes and gene evolution of the PaLoc and CdtLoc is an important initial step to define expected disease progression and provide the most relevant clinical response. However, due to many other components of the CDI life cycle, the toxinotype does not always correlate in a predictable manner with disease severity or the epidemic nature of the CDI.

## 3. CDT Structure and Mechanism of Action

The CDTa and CDTb subunits of CDT show an 81% and 84% amino acid sequence identity to *Clostridium perfringens* iota toxin Ia and Ib, as well as 80% and 82% identity to *Clostridium spiroforme* Sa and Sb, respectively [8,14,15,16]. Thus, it was not surprising that the CDTa and CDTb subunits of CDT are interchangeable with subunits from *C. perfringens* iota toxin and *C. spiroforme* toxins and thus display overlap of their evolutionary pathways [17]. Likewise, host cell entry and cytotoxicity from CDT occurs in a synergistic manner similar to that of other binary toxins, such as *C. perfringens* iota toxin, *Clostridium botulinum* C2 toxin, *Bacillus anthracis* edema and lethal toxin, and *C. spiroforme* toxin [15]. Like CDT, these other binary toxins possess an intact ‘A-B’ structure having an enzymatic component (A) and a host cell binding or pore-forming component (B) [18,19]. Upon proteolytic cleavage of a pro-domain sequence, the ‘B’ components assemble into oligomeric structural units together with the membrane-bound host cell-surface receptor. Likewise, the ‘A’ subunit binds to the ‘B’ oligomer complex in all binary toxins prior to being endocytosed and trafficked into endosomes. While the ‘A’ subunit is monomeric for each binary toxin, the oligomeric structure has been shown to consist of varying compositions including heptameric, octameric, as well as di-heptamer associations of singly processed ‘B’ subunits [19,20]. Likewise, it is hypothesized that a conformational shift in the ‘B’ subunit assembly, facilitated by the acidic pH of the endosomal compartment, induces translocation of the ‘A’ subunit into the host cytosol where the host NAD^+^/NADPH acts as a donor for catalytic transfer of ADP-ribose to monomeric G-actin. This covalent modification to G-actin prevents its proper polymerization as well as its normal association with F-actin. Such disruption of the G-actin:F-actin equilibrium destroys the integrity of the host cell cytoskeleton and leads to rapid cell death [6,21].

### 3.1. Structure of the CDTa Enzymatic Subunit

**Figure 2:**
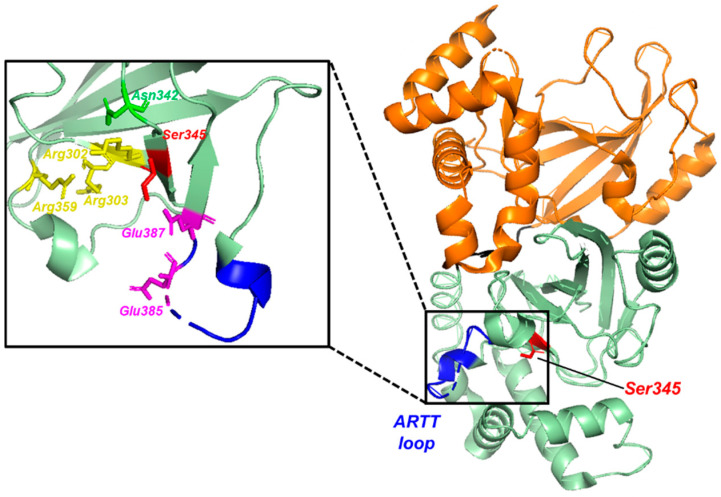
X-ray Crystal Structure of CDTa modified from PDB file: 2WN8. The N-terminal region (residues 1–215, orange) is the CDTb-binding domain. The C-terminal region (residues 224–420, pale green) is the catalytic domain with ADP-ribosyltransferase activity. The ARTT loop (residues 377–387, blue) is crucial for substrate binding and ADP-ribosylation as the active site residues are located around this region (inset). However, Glu385 and Glu387 in the ARTT loop are not directly involved in the catalytic site formation. A small peptide loop (residues 216–223, black) connects both termini.

Unprocessed CDTa has an unstructured region that may be important for CDT assembly and/or its host cell entry based on studies with CDT and other binary toxins, including anthrax and iota toxins [22,23,24]. Removal of this N-terminal 43 amino acid signaling sequence results in the ~47.5 kDa mature and fully toxic CDTa enzyme. Acharya and coworkers first reported X-ray crystal structures of mature CDTa at several pH values including 4.0, 8.5, and 9.0, and some minor structural variations were observed [25]. In each case, CDTa was found to be a two-domain enzyme with each domain adopting an α/β fold having five α-helixes aligned perpendicularly to an eight stranded β-sheet (Figure 2). The two α/β domains are connected by a flexible loop (residues 216–223) with each domain having a unique amino acid sequence as needed for the toxin’s molecular mechanism of action. As with other binary toxins, the N-terminal α/β domain of CDTa (^N-term^CDTa residues 1–215) is important for CDTa binding to CDTb; whereas residues important for the enzyme’s catalytic toxicity reside in a cleft of the C-terminal α/β domain (^C-term^CDTa; residues 224–420). That an isolated ^N-term^CDTa construct can compete with full-length CDTa for binding to iota toxin binding component, Ib, further supports the notion that iota toxin, CDT, along with other binary toxins have functionally conserved structural domains [17]. Furthermore, the isolated ^N-term^CDTa domain was found by NMR to remain structurally intact throughout the entire α/β domain fold based on comparisons of its secondary structure to that of the full-length enzyme (PDB: 2WN4) [25,26]. The ^N-term^CDTa domain (residues 1–226) was further demonstrated to be functionally intact with regard to its cargo delivery capacity since engineering this ^N-term^CDTa α/β domain to link to the catalytic component of the large toxin, TcdB, was sufficient to deliver the glucosyltransferase activity of the large toxin into mammalian cells [27]. These data together show that the CDTb binding component of CDTa, ^N-term^CDTa, could likely be used as a biologically efficient intracellular delivery system, in a general way, for other protein engineering experiments.

The ADP-ribose moiety of host cell coenzymes NAD^+^ and/or NADPH are catalytically transferred by the enzymatic subunit of the binary toxin to a specific arginine residue in G-actin of the host cell. As with other binary toxins, the second α/β domain of CDTa, ^C-term^CDTa, has a well-defined cleft that harbors residues important for this ADP ribosylation activity [25]. For example, Arg296, Arg352, Glu378, Glu380, Gln300, and Asn335 important for iota toxin catalytic activity coincide with the Arg302, Arg303, Arg359, Glu385, Glu387, Gln307, and Asn342 required for CDTa catalytic activity [25,28,29]. Despite such structural similarities, the acidic residue (Glu387) in CDTa, analogous to Glu380 in Ia, is not detected as an interacting site for the coordination of NAD^+^ or NADPH. Hence, subtle differences are found to occur when binary toxins are compared, particularly in a region termed the “ADP-ribosyl turn-turn” loop (ARTT loop).

The ARTT loop is found in most ADP-ribosylating toxins, including CDT, cholera toxin, diphtheria toxin, and *E. coli* enterotoxin [30]. Although the length of the loop varies, it contains residues that are important both for substrate binding and for the enzyme’s ADP-ribosylation activity. The ARTT loop in CDTa (Figure 2) includes residues 377–387, and fast timescale dynamics observed by NMR for several residues in this loop, shows that they are highly flexible, a feature that is likely important for substrate binding to CDTa as well as for its catalytic activity [31]. Such a conclusion is supported by studies showing that NAD^+^ or NADPH binding to CDTa stabilized the disordered character of the ARTT loop (unpublished observation). Other conditions, including pH, were also found to change the structural characterization of this loop. For example, at basic pH values between 8.5 and 9.0, a stable loop structure was observed while lowering the pH to 4.0 caused residues within the ARTT loop to be unobservable via X-ray crystallography and were likely dynamic in nature [25]. Likewise, the NAD^+^/NADPH substrates were found to form hydrogen bonds with Arg302, Arg303, Arg359, Gln307, Asn342, and Ser345, at basic pH values, also implicating the importance of these residues in the enzyme’s active site (Figure 2, inset) [25]. The importance of specific residues for ADP-ribosylation activity was examined further using site-directed mutagenesis [32]. Specifically, the S345A mutation was found to block cellular toxicity as monitored using Vero cell death while the S435Q, S345F, and S345R mutations abolished any detectable activity (>1000-fold) [32]. Because these mutations at S345 did not affect substrate binding directly, it was concluded that S345 was important for the enzyme’s catalytic ribosyl transferase activity.

Additional mutagenesis studies of active site residues confirmed that residues S345, E385, and E387 were important for CDTa catalytic activity [33,34,35,36]. Likewise, a triple mutant, 3mCDTa (S345F+E385Q+E387Q) and a quadruple mutant, 4mCDTa (C2A+S345F+E385Q+E387Q) completely inhibited the cellular cytotoxicity (>1000-fold) associated with CDT [33,36], but remained folded as determined by NMR as well as by studies showing that the mutant constructs both acted as potent dominant negative constructs on their own [33]. It is worth noting that a C2A mutation was introduced for experimental purposes to eliminate residual dimerization but had no effect on enzymatic activity on its own [36]. Lastly, the 4mCDTa tetravalent mutant served as a potent antigen in immunology studies in which inoculating rodents with this construct provided a safe protective efficacy in vivo from the 027/B1/NAP1 strain of *C. difficile* [34].

### 3.2. Structure of the Binding Domain

**Figure 3:**
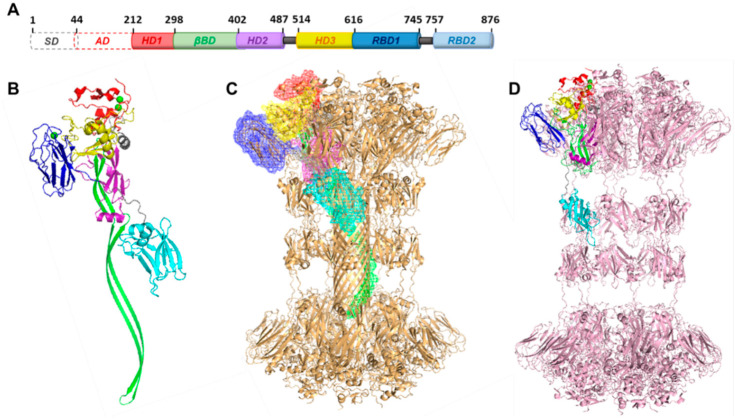
Structure of CDTb. (**A**) The sequence of the pro-CDTb before proteolytic cleavage of signaling domain (SD) and activation domain (AD). Activated CDTb consists of residues 212–876. (**B**) Structure of activated ^Asym^CDTb monomer corresponding to each domain in the sequence; HD1 (red), βBD (green), HD2 (purple), HD3 (yellow), RBD1 (blue), and RBD2 (cyan). Linker regions 1 and 2 are shown in grey and the green spheres represent Ca^2+^ ions in HD1 and RBD1. (**C**) Structure of activated ^Asym^CDTb di-heptameric assembly highlighting the arrangement of individual domains of the monomer. ^Asym^CDTb structure is modified from PDB file: 6UWR. (**D**) Structure of activated ^Sym^CDTb di-heptameric assembly with corresponding domains of the monomer. The βBD is not elongated to represent the pore-forming unit and therefore ^Sym^CDTb is identified as a pre-pore state. ^Sym^CDTb structure is modified from PDB file: 6UWT.

Pro-CDTb (residues 1–876) is an inactive monomeric precursor of the CDTb cell binding and pore-forming component of the CDT binary toxin. Limited chymotrypsin proteolysis of pro-CDTb is sufficient to remove the signaling peptide (SD; residues 1–43) and the activation domain (AD; residues 44–211) yielding an oligomeric and active form of CDTb (residues 212–876; Figure 3A,B) [15]. Unlike the heptameric and octomeric states identified for the cell-binding components of other bacterial binary toxins such as anthrax and iota toxins, CDTb was shown by single-particle cryoEM micrsoscopy to fold into two unique di-heptameric structures (~1.0 MDa) including a symmetric and an asymmetric dimer of heptamers, solved at resolutions of 3.14 Å (^Sym^CDTb) and 2.84 Å (^Asym^CDTb), respectively [20]. A corroborative study confirmed these data at a lower resolution and were given names as ‘short’ and ‘long’ particles, corresponding to the asymmetric (^Asym^CDTb) and symmetric (^Sym^CDTb) di-heptamers, respectively (Figure 3C and 3D) [37]. In both cryoEM studies, the di-heptamer fold showed a central donut-like structure from a top-view with what looks like an “open” and “closed” pore and dumbbell-like structures from the side-views with differing lengths with ^Sym^CDTb structure being the longer of the two structures.

The extended β-barrel assembly in the ^Asym^CDTb structure (Figure 3C), which is also known as the ‘pore-state,’ resembles the membrane-inserted structure identified for anthrax toxin [38]. The ^Asym^CDTb structure can be formed in the absence of a lipid bilayer or detergents; therefore, it is identified as a relatively stable entity. In the ^Sym^CDTb form (Figure 3D), the two identical heptamer domains lack the extended β-barrel and it represents a pre-pore state of the binary toxin. Detailed analysis of the structure (Figure 3A,B) depicted the presence of different domains including a heptamerization domain (HD1; residues 212–297), a β binding domain (βBD; residues 298–401), a second heptamerization domain (HD2; residues 402–486), a linker region (L1; residues 487–513), a third heptamerization domain (HD3; residues 514–615), a receptor-binding domain (RBD1; residues 616–744), a second linker (L2; residues 745–756), and a second receptor-binding domain (RBD2; residues 757–876) [20]. These domains also correspond to the different moieties identified by the Lacy group; D1, pore-forming loop (PFL), D2, linker, D3, D3′, linker, and D4, respectively [37]. The RBD1 domain represents a discrete non-homologous structure that was not identified in other binary toxins and the RBD2 domain possesses no sequence homology to other toxins except to some degree for the iota toxin.

The heptamerization domains identified in the core unit depict similar folds observed for other clostridial toxins such as iota toxin and anthrax toxin [39]. Notably, Ca^2+^-coordinating sites were discovered for the HD1 domains residing in both ^Asym^CDTb and ^Sym^CDTb [20], and analogous calcium ion binding sites were recognized prevously in the HD1 domains of anthrax toxin, iota toxin, as well as being highly conserved throughout this toxin family [40,41]. For anthrax, the role of calcium ions was found to be necessary for both the structural stability and proteolytic activity of the anthrax toxin [40,42,43]. In addition to structural calcium ions, extracellular calcium levels are also deemed essential for endocytosis during the intoxication pathway, as calcium depletion was shown effective for protection against anthrax toxin cell penetration [44]. Likewise, the coupling of Ia and Ib, and subsequent cytotoxicity of the iota toxin are dependent on extracellular calcium [41]. Thus, potent calcium-chelating agents were not surprisingly shown to neutralize CDT activity [45].

The β-barrel heptameric unit is composed of double-stranded antiparallel β-sheets, with several hydrophobic residues at the tip of the β-barrel that can potentially stabilize CDTb insertion into the membrane by protecting it from solvent exposure [20]. Structural comparison of the heptameric core of the ^Asym^CDTb and ^Sym^CDTb units revealed drastic changes in the residues contributing to the β-barrel formation. In the ^Sym^CDTb structure, residues corresponding to the elongated barrel assembly in ^Asym^CDTb form a four-stranded antiparallel β-sheet that packs between the HD3 and RBD1 domains. Interestingly, the unique structural changes in the β-barrel assembly of ^Asym^CDTb can contribute to the reorientation of the phenylalanine residue (Phe455) in HD2 to facilitate the functionally significant φ-gate unit. The φ-gate is essential for the translocation of CDTa through the CDTb pore [38]. Similar φ-gate conformations are conserved in Ib and protective agent (PA) of anthrax toxin as translocation channels [23,38]. Recently, it was reported that a point mutation at Phe454 in the iota toxin, which is the complementary residue to Phe455 in CDTb, resulted in the full loss of cytotoxicity [23]. Additionally, the structural elucidation of the iota toxin revealed that translocation of Ia is propagated by the free accessibility of the hydrophobic φ-gate for the unfolded N-terminal residues of Ia.

The RBD1 domain is structurally unique to CDTb, as no distinct similarity has been reported with the corresponding binary toxin structures. The discrete structure of the RBD1 domain is composed of a 10-stranded β-sandwich. Structural exploration by a distance matrix alignment (DALI) query suggested that RBD1 has similar features of glycan-binding modules [37]. Glycan-array screening conducted with the CDTb D3′ domain, which is analogous to the RBD1, has demonstrated positive interactions with several carbohydrates such as fucose, L-rhamnose, chitobiose, chitotriose, and lacto-N-tetraose [37]. Specifically, the presence of excess fucose and chitobiose caused a slight increase in monolayer disruption of the CDT toxin favoring the activity. Although carbohydrate binding of similar binary toxins is not well-established, carbohydrates like N-acetylglucosamine were identified as important components for cellular binding and cytotoxic capacity of *C. botulinum* C2 toxin [46]. Typically, the β-sandwich carbohydrate-binding modules are metalloproteins and the ligand recognition by many of these domains is calcium-dependent [47]. Interestingly, a unique calcium-binding site within the RBD1 domain of the X-ray crystal structure of ^Asym^CDTb was observed, and when this RBD1 domain was isolated (residues 616–744), its stability and structural fold was dependent on the presence of calcium, as determined by NMR [20].

The second RBD is connected to RBD1 with a short linker, and the structural features of RBD2 were not identified in other members of this toxin family. In addition, the location of RBD2 is different when compared to the structures of ^Asym^CDTb and ^Sym^CDTb and this results from the long β-barrel feature of the ^Asym^CDTb state [20]. Since the deletion of RBD2 in CDTb resulted in a heptameric structure with significantly reduced cytotoxicity, it was postulated that the RBD2 domain is crucial for the di-heptamer assembly as well as being critical for delivering toxic CDTa to host cells. Moreover, DALI structural exploration implied that RBD2 remotely resembles the receptor-type protein tyrosine phosphatases and other receptor-like proteins promoting cell adhesion and ligand binding. Interestingly, CD45 which is a similar glycoprotein to CD44, a cell receptor of CDTb, discussed in detail below, is a prototypical receptor-type protein tyrosine phosphatase [48]. These subtle yet key points in the toxin structure facilitate a better understanding of the intoxication machinery and will help to aid with its effective inhibition.

## 4. Receptor Interaction and a Plausible Molecular Mechanism

**Figure 4:**
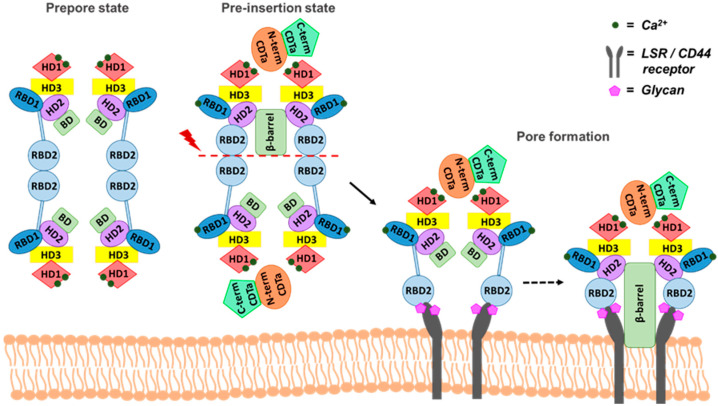
The putative mechanism for cell entry of CDT. ^Sym^CDTb can acts as a pre-pore state while ^Asym^CDTb is a potential pre-insertion state. The binding of CDTa to CDTb via the N-terminal region may trigger the decoupling of heptameric units. The resulting CDT complex can engage with the cell surface receptors (LSR or CD44) embedded in the cell membrane, localized to lipid rafts, and this phenomenon is potentially facilitated by glycan binding.

The structure of the binding component, CDTb, is often compared to heptameric and octameric structures of the iota toxin family, as well as PA of the anthrax toxin. Therefore, the mode of action of CDT is hypothesized to mimic this same mechanism of action. However, the discovery of the di-heptamer unit challenges this idea and hints that subtle changes in the mechanism may facilitate the β-barrel pore formation in the cell membrane. One hypothesis is that ^Sym^CDTb is a pre-pore state while ^Asym^CDTb is a pre-insertion state due to the presence of this β-barrel structure. However, a clear understanding of the cellular pathway that triggers the separation of two heptameric units is lacking, although interaction with the host-cell receptor and/or membrane is likely involved (Figure 4).

The lipolysis-stimulated lipoprotein receptor (LSR) and the cluster of differentiation 44 protein (CD44) are identified as two main membrane-bound host cell receptors that can interact with CDTb (Figure 4). LSR is composed of an extracellular immunoglobulin-like domain, a short transmembrane helix, and a long cysteine-rich intracellular domain [49]. This type I single-pass transmembrane protein of 581 amino acids is highly expressed in the liver and other tissues, such as the small intestine, colon, kidney, and lung [50]. Although LSR belongs to a family of the immunoglobulin-like domain containing receptors, which are involved in the formation of tricellular tight junctions upon cell contact of epithelial cells, only LSR functions as a receptor for CDT [51]. Notably, LSR also acts as the cellular receptor for other binary toxins such as C. perfringens iota toxin and C. spiroforme toxin, which have almost 90% sequence homology in their respective binding domains to CDTb.

LSR was first identified as the target-cell receptor of CDT by a genome-wide haploid genetic screen [52]. The direct receptor interaction of CDTb is emphasized by a pull-down assay conducted with the GST-tagged soluble extracellular domain of LSR. Parallel studies provided evidence for a required interaction of *C. perfringens* iota toxin with LSR to enter the cytosol. However, *C. botulinum* C2 toxin, which has only a 12% sequence similarity to the C-terminal receptor-binding domain of the CDTb (specifically RBD2), shared no direct binding to LSR for cell entry. More specifically, direct binding of CDTb occurs at the Ig-like domain of LSR, and a binding affinity of ~110 nM was reported from surface plasmon resonance (SPR) measurements [51]. Coincidently, the Ig-like domain is also present in the anthrax toxin receptor 1. However, the anthrax toxin shares no interaction with the Ig-like domain and thus identifies the subtle differences in the toxin behavior due to structural disparities. Moreover, the LSR binding site of CDTb resides in the region of amino acid 757–866 which represents the RBD2 domain. Papatheodorou *et al.* identified this binding region by generating GST-fused proteins with N- and C-terminal truncations of RBD [52]. In addition, glycan sugars in the extracellular environment are thought to be needed to facilitate the RBD2 -receptor interaction [37].

CDTb binding to LSR was found to trigger the accumulation of LSR into detergent-resistant membranes, which are sub-compartments of the plasma membrane containing typical markers of the lipid rafts [53]. The lipid rafts were also recognized to mediate cell entry for iota-like toxins since iota toxin pre-pore conformations were found to be associated with cholesterol-rich detergent-resistant membrane microdomains [54,55]. Likewise, depletion of membrane cholesterol was found to prevent Ib oligomerization and blocked the cellular toxicity of the binary toxin. Interestingly, pro-CDTb was shown to propell the clustering of LSR into lipid rafts and thus enhancing the argument that lipid rafts are an important component of CDT cellular entry as well as indicate that monomeric pro-CDTb is also could play a role in CDT entry at the host cell receptor-membrane interface [53]. More recently, Lucy and co-workers reported that one CDTa molecule is bound to a heptamer of CDTb by cryoEM in the presence of the LSR extracellular domain. This structure provides evidence that the LSR domain binding to CDTb is sufficient to dissociates the di-heptamer and could represent what they term the “pre-pore state” of CDT. However, this cryoEM structure was solved using a truncated version of the CDTa (^Δ(1−17)^CDTa), so whether this structure is physiologically relevant remains to be shown.

Coincidently, the cell surface receptor for hyaluronic acid, CD44, was also identified in the lipid rafts [56] with a three-fold upregulation of CD44 found in the detergent-resistant membrane proteome of Vero cells upon treatment with Ib [57]. These data together with the discovery that CD44 expression was sufficient to induce toxicity via CDT and other iota-family binary toxins provided evidence that this host-cell receptor represented another means for binary toxins to enter host cells was discovered to impart intoxication upon binding to CDT and other iota-family binary toxins [58]. That CD44-deficient cell lines showed resistance to CDT toxicity further strengthened this notion as did pull-down experiments, which confirmed direct binding of CDT to the CD44 receptor [58].

After host cell receptor binding at the membrane surface, the current hypothesis for CDT delivery into the cytoplasm is thought to involve a potential conformational change in CDTb at the low pH of the endosomal compartment [49]. There is also intriguing evidence that translocation of the iota toxin occurs via a membrane potential gradient, which may be a prospective aid in the CDT entry mechanism [59]. Following translocation into the cytosol, Arg177 of monomeric G-actin is irreversibly ADP-ribosylated by CDTa [60] causing eventual depolymerization of F-actin and destruction of the cytoskeleton due to the disorganization of microtubules. Actin depolymerization promotes the propagation of the long membrane protrusions initiated by the growth of microtubule plus ends [49]. These microtubule-based protrusions develop into a tentacle-like network on the surface of epithelial cells to enhance the adherence of Clostridia [18,61,62]. In addition, the occurrence of these highly dynamic protrusions depends on cholesterol-rich lipid microdomains, which are associated with the membrane receptors of CDT [62]. Together, this complex mixture of CDT subunits, host-cell receptors, and cholesterol-rich membrane are required for CDT to enter and kill host cells. Thus, all of these biological components should be considered in strategies to target toxicities associated with the binary toxin, CDT.

## 5. Concluding Remarks

Over the past two decades, substantial progress was made towards unraveling the cellular uptake mechanisms of the ADP-ribosylating binary toxins. The recent discoveries of distinctive structural arrangements in CDT provide several surprising findings that now demand further clarification of the mechanism of action leading to CDI pathology. Such advances in both structural and functional studies of the *C. difficile* binary toxin can now be used for developing immunization and drug targeting strategies for clinical purposes.
